# Informing women about hormone replacement therapy: the consensus conference statement

**DOI:** 10.1186/1472-6874-9-14

**Published:** 2009-05-29

**Authors:** Paola Mosconi, Serena Donati, Cinzia Colombo, Alfonso Mele, Alessandro Liberati, Roberto Satolli, Consensus Conference Working Group

**Affiliations:** 1 Laboratory for medical research and consumer involvement, Istituto di Ricerche Farmacologiche Mario Negri, Via La Masa 19, 20156 Milan, Italy; 2 Reparto Salute della donna e dell'età evolutiva-CNESPS, Istituto Superiore di Sanità, Viale Regina Elena 299, 00161 Roma, Italy; 3 Epidemiologia Clinica e Linee Guida-CNESPS, Istituto Superiore Sanità, Viale Regina Elena 299, 00161 Roma, Italy; 4 Dept of Haematology, Oncology and Respiratory Disease (University of Modena and Reggio Emilia) Director Italian Cochrane Centre, Istituto di Ricerche Farmacologiche Mario Negri, Via La Masa 19, 20156 Milan, Italy; 5 Zadig, Scientific publishing company, Via Calzecchi 10, 20133 Milan, Italy

## Abstract

**Background:**

The risks/benefits balance of hormone replacement therapy is controversial. Information can influence consumers' knowledge and behavior; research findings about hormone replacement therapy are uncertain and the messages provided by the media are of poor quality and incomplete, preventing a fully informed decision making process.

We therefore felt that an explicit, rigorous and structured assessment of the information needs on this issue was urgent and we opted for the organisation of a national consensus conference (CC) to assess the current status of the quality of information on hormone replacement therapy (HRT) and re-visit recent research findings on its risks/benefits.

**Methods:**

We chose a structured approach based on the traditional CC method combined with a structured preparatory work supervised by an organising committee (OC) and a scientific board (SB). The OC and SB chose the members of the CC's jury and appointed three multidisciplinary working groups (MWG) which were asked to review clinical issues and different aspects of the quality of information. Before the CC, the three MWGs carried out: a literature review on the risk/benefit profile of HRT and two surveys on the quality of information on lay press and booklets targeted to women. A population survey on women's knowledge, attitude and practice was also carried out. The jury received the documents in advance, listened the presentations during the two-day meeting of the CCs, met immediately after in a closed-door meeting and prepared the final document. Participants were researchers, clinicians, journalists as well as consumers' representatives.

**Results:**

Key messages in the CC's deliberation were: a) women need to be fully informed about the transient nature of menopausal symptoms, about HRT risks and benefits and about the availability of non-pharmacological interventions; b) HRT is not recommended to prevent menopausal symptoms; c) the term "HRT" is misleading and "post menopausal hormone therapy" should be the preferred definition.

**Conclusion:**

This CC led to the identification of specific information drawbacks. Women are exposed to messages that are often partial, non evidence-based nor transparently developed. The structured and participative methodology of this CC allowed a multidisciplinary perspective and a substantial lay people input.

## Background

In Italy, as in other countries, opinions and views about hormone replacement therapy (HRT) vary widely among scientific societies, specialists, and general practitioners [1].

At the beginning of the nineties, observational studies suggested that HRT was effective for most symptoms and diseases of menopausal women and its use was recommended as the intervention was deemed to reduce cardiovascular and osteoporotic risks. However, HRT's disadvantages emerged from two large randomized controlled trials (RCTs) – the Heart estrogen-progestin replacement study (HERS) [2] and the Women health initiative (WHI) [3] – that found no benefits for prevention of cardiovascular diseases. Particularly, the WHI trial found an increase in breast cancer incidence and in cardiovascular events in healthy women. Later, The million women study [4] showed an increase in breast cancer incidence associated with the therapy. The publication of these new findings, supported by large RCTs, led to different interpretations among researchers, specialists (mainly gynecologists and oncologists) and general practitioners. The subsequent guidelines and recommendations endorsed by Italian medical societies and healthcare institutions often disagreed. Not surprisingly, also patients and consumers associations' documents, booklets and brochures gave contradictory messages. In this context, information on menopause was often incorrectly reported: it is described as a disease, especially in newspapers and TV programs, leading to the medicalisation of a natural event in women's life. For all these reasons, women are likely to receive unbalanced and incomplete information from doctors, mass media and other sources and confusion prevails on the pros and cons of taking hormones for menopausal symptoms.

We therefore felt that an explicit, rigorous and structured assessment of the information needs on this issue was urgent and we opted for the organisation of a national consensus conference (CC) aimed at assessing the correspondence/dissonance between the state of art of scientific research and the information that is conveyed to women. Besides the traditional format of a CC – including a two-day meeting with a jury listening to the reports of expert witnesses and then issuing a set of recommendations – we set up a process, already tested in previous CCs in Italy, whereby the two-day meeting comes at the end of highly structured and intense preparatory activities which are described in details below. The conference was entitled "Informing women about hormone replacement therapy" and it was organised by the Partecipasalute project – coordinated by Istituto di Ricerche Farmacologiche Mario Negri with the collaboration of Italian Cochrane Centre and Zadig, scientific publishing company – and the National Guidelines System (SNLG) based at the Istituto Superiore di Sanità. The conference took place in Turin on 16 and 17 May 2008.

## Methods

In April 2007 the organising committee (OC) identified a scientific board (SB) to support the development of the conference and, together with the SB, appointed a multidisciplinary jury. The OC and the SB, after discussing different aspects of the issue, defined the main questions to which the CC jury was called to answer (table 1). Members of the scientific board, the jury and the working groups are listed in appendix 1. Ethics approval was not necessary for CC.

**Table 1:** Questions addressed by the consensus conference

**1**.	What is the information about menopause and HRT that women would value most?
**2**.	What is the average quality of the information available on this health issue? Which are the main risks for the general public and professionals associated with the dissemination of deceitful and unreliable information? How to improve the scientific quality of the information?
**3**.	Which characteristics of the menopause can be linked to health problems?
**4**.	Why should we prescribe HRT, which are the main objectives of the therapy, which women are eligible to receive it and what would be the duration of the treatment?
**5**.	Which alternatives to HRT, especially non-pharmacological, need more information?
**6**.	What are the priorities for future research?

### Working groups

In order to weigh up the scientific data available on HRT and to evaluate the information conveyed to women from different sources, three working groups were appointed, dealing with: scientific evidence available; information published in newspapers and magazines; information provided in booklets, brochures and websites specifically targeted to women and focused on menopause. The first group included two gynecologists, two general practitioners, one cardiologist and one endocrinologist. This working group assessed the scientific literature available on HRT starting from guidelines published by public institutions, then Cochrane reviews or meta-analysis, individual primary studies directly assessing the impact of HRT on women's health and, finally, observational studies or statements made by experts. This latter source was considered only if the method to produce the documents was described and potential conflicts of interests disclosed.

The working group dealing with information published in the press was composed of five journalists and one clinician; the other group, dealing with booklets, brochures and information material targeted to women was composed of patients' associations representatives, researchers and lay members of ethics committees. Two hundred and twenty five articles published in the press from 2000 to 2007 and 78 documents targeted to women were extracted from available sources (publisher's archives or search engines in internet) and appraised using a standardized form dealing with graphic layout, editing, transparency of information, contents related to menopause and HRT. Specifically, the main items of the form were: date of publication; title of the document; ownership of the document or website; author; sponsorship; presence of photos or tables; length of the document; type of information conveyed; sources of information; how menopause is described (as a disease, as a physiological phase); how HRT is described: is it suggested to cure symptoms? To prevent risks? Are HRT's risks described? Is the length of therapy reported? Are treatments alternative to HRT reported? Finally, clearness of the language used.

All the working groups worked independently, according to self-defined explicit criteria, supported in their organisation by the OC.

### Population survey

A survey was carried out by the Istituto Superiore di Sanità to seek information on women knowledge, attitudes and behaviors about menopause and HRT. The survey involved representative samples of women 45–60 years from 5 Italian Regions, a total of 969 women were selected from electoral rolls. Seven hundred twenty of them (74,3% response rate) completed the interview carried out by trained midwifes and social workers of maternal and child health centers. The questionnaire investigates women's experience in relation to menopause (quality and prevalence of symptoms) hormone therapy (use, indications, prescription and length of use) and other treatments used; the type and amount of information received about menopause, hormone therapy and other possible treatments; finally, it gathers information about personal habits, health status and socio-demographic characteristics [5].

### Declaration of interest

In preparation of the CC, the OC made several efforts to involve in the CC's process all interested parties and stakeholders. An announcement was published in the Partecipasalute and SNLG websites (; ) and e-mails were sent to medical societies, pharmaceutical companies, patients' associations and other potentially relevant institutions. The announcement invited interested groups to submit documents, comments or requests to participate at the public final session of the CC. We collected thirty-three declarations of interest in the conference and twenty-eight documents were submitted to the OC. All the documents collected were assigned to one of the three working groups, according to the type of issue dealt with (scientific articles or documents; information issues; booklets targeted to women).

### Conflict of interests

All members of the working groups, the scientific board and the jury signed a declaration about their conflict of interests.

### Jury

The CC's jury was chaired by a lay member and was composed of a member of the National agency for regional healthcare services, two consumers' representatives, a bioethicist, a jurist, two journalists, a gynaecologist, two general practitioners, a clinical pharmacologist, an obstetrician, a psychotherapist. The jury worked in complete autonomy from the OC. It set its own procedural rules, accepted by members 15 days before the public session as a prerequisite to participate to the jury's work. The jury had access to the documents produced by the working groups one month before the public session of the CC. During the public session in Turin, the jury listened to the oral presentations of the working groups' data and then, behind closed doors, it discussed the documents received and defined the consensus statement. The preliminary version of the consensus document was presented to the public and the media. The writing committee, appointed by the jury from within its members, completed the document, without any substantive modifications of the conclusions agreed by the whole group. The final document was approved by the jury one month after the two-day open meeting.

## Results

The CC's final document (see Additional file 1: Final Report CC-HRT.pdf) was prepared taking into account all the data and documentation produced by the three working groups and the discussion had during the two-day open meeting in Torino. The document has been produced for the general public, medical and non-medical experts interested in this issue or working in the area of menopause and HRT. The jury used two different approaches to communicate the main messages: 1) a concise writing style to improve the clarity and dissemination of the information; 2) a specialist and scientific style, more accessible to medical professionals.

The jury considers the term "hormonal replacement therapy" (HRT) as misleading since it may imply a situation of hormonal deficiency and, consequently, a diseased state. Since this concept is not in line with the one being used in the final document, the jury recommends instead the term post-menopausal hormonal therapy (PHT) as more appropriate. However, the term HRT has been used in the document and the terminology has not been amended, since the document answers to questions formulated and defined with specific terms by the organising committee and, consequently, not modifiable.

According with the questions of the consensus conference, the results fit in three major areas of interest: information issues, clinical issues, and priorities for future research.

### Information issues

The data collected by the two working groups dealing with information are summarised in table 2. They were evaluated by the jury in order to answer the questions defined by the OC, together with the population survey's results reported elsewhere [5].

**Table 2:** Summary results of information working groups

**Working groups dealing with information targeted to women (brochures, etc...)**
Documents selected and reviewed: 78, most of them were published online
* 13 documents published by the national health system (NHS), 11 by scientific societies and 54 by private sources (pharmaceutical companies, publishing houses and patients associations)
* 68–87% of documents, particularly published online, were poorly written and had an inadequate graphical quality (13% reported tables or graphs and 32% illustrated with photos or images)
* less than 50% described menopause as a physiological phase of the life
* about 33% described the problems related to menopause as a disease
* about 75% of the pharmaceutical and specialised documents, and 50% of the documents produced by the NHS and scientific societies described HRT as a preventative measure; the main indication to HRT is the prevention of bone fractures
* 60–80% of the documents published by scientific societies, NHS, publishing houses, patients' associations, and in less than 50% of pharmaceutical and specialised documents reported the risks associated with HRT. Breast cancer is the mainly reported risk
* 50% of the documents were linked to commercial activities or sponsors, particularly herbal products
* 30–50% of documents did not report the original sources of information and 20% of the articles were written by an expert in the field
* 51% of the documents did not report information on sponsorship
**Working group dealing with information published in mass media**
Documents selected and reviewed: 225 articles
* 20 articles published in women magazines, 69 health magazines, 64 weekly news magazines, 32 newspapers, and 40 specialised journals
* most of the articles included pictures of women much younger than the HRT target Women
* 68% referred to the opinions of an expert in the field as the main sources of Information
* HRT is recommended to cure symptoms mainly in specialised journals (63%), and weekly news magazines (61%)
* 15% women magazines, 49% health magazines, 52% weekly news magazines, 38% newspapers and 68% specialised journals described HRT as a preventative measure
* 60% of the articles cited the risks of HRT
* the working group concluded that about half of the articles published in weekly news magazines and specialised periodicals, a fourth of the articles published in women's magazines and less than a third in health magazines might be helpful for women to make informed decisions
* the information on conflicts of interest is missing in 88% of the articles

What is the information about menopause and HRT that women would value most?

The information provided to women and to health operators should always be independent and evidence-based, dealing with:

• the concept of menopause as part of the natural life cycle;

• symptoms strictly related to the menopause, their duration and the available treatments;

• risks and benefits of the treatments available (pharmacological and alternative therapies);

• useful lifestyle changes.

Every woman should be able to exercise her right to take a conscious and informed decision, after having access to qualified sources of information able to provide definite answers to questions related to menopause, therapies, risks, uncertainties still present in research, and possible non-pharmacological treatments.

What is the average quality of the information available on this health issue? Which are the main risks for the general public and for professionals associated with the dissemination of deceitful and unreliable information? How to improve the scientific quality of the information?

The first articles published about HRT have initially highlighted its benefits. Subsequently these findings were revised by studies published in the medical literature, which have characterized the risks associated with the treatment. The overall quality of the information about menopause (from professional and non-professional sources) is poor and it has clear methodological flaws, contradictions and conflicts of interests (economic and professional) [6]. Actually, the risk related to this misinformation is that women with non tolerated menopausal symptoms would be prevented from having HRT even though they could benefit from the treatment with relatively modest risks. Therefore, it is critical to deliver scientific and correct information on the current knowledge, advantages and disadvantages, including areas of uncertainties, pharmacological and non-pharmacological alternative treatments and their effectiveness.

The information should neither promote the use of HRT nor raise concern about the use of HRT. Every therapeutic decision should be based on an empathic patient-doctor relationship to take into account the preferences and priorities of every patient.

### Clinical issues

Which characteristics of the menopause can be linked to health problems?

Menopause is one of physiological phases in women's life, which could sometimes affect their quality of life.

The medical problems with a proved association with menopause are:

• vasomotor symptoms (sweating, flushing)

• vaginal dryness

• disturbed sleep.

Vasomotor symptoms and disturbed sleep are generally short-lasting and variable in their intensity; however, they may significantly affect women's quality of life. Other medical conditions, frequently associated with menopause (irritability, depression, osteoarticular pain, weight gain) do not have a causal link with it, but they would similarly require medical attention.

Why should we prescribe hormonal replacement therapy (HRT), which are the main objectives of the therapy, which women are eligible to receive it and what would be the duration of the treatment?

Hormonal replacement therapy should be prescribed to women with an early onset of the menopause, considered as a pathological state, and to women with vasomotor problems and disturbed sleep perceived as important and persistent [7]. Vaginal dryness and difficult sexual intercourse (dyspareunia) are not considered criteria for the recommendation of systemic HRT and they can be treated with topical treatments, which are generally effective.

However, women with milder symptoms may have a negative perception of the menopause and therefore they may explicitly request HRT. The criteria for the prescription of HRT in these cases cannot be defined and generalized and the therapeutic plan for each patient should be individually discussed with a medical doctor.

Women should be informed that menopausal symptoms may be short-lasting (with the exception of vaginal dryness) and benign and they should be aware of the risks and benefits associated with the use of HRT, of the frequent recurrence of the symptoms after its interruption and of the availability of non-pharmacological treatments able to alleviate symptoms in order to make an informed and conscious decision. Every woman should receive updated and clear information on the available non-pharmacological treatments and lifestyle modifications programs.

Clear evidence about the optimal duration of the treatment to effectively control the symptoms is currently not available. The approved clinical guidelines, which specifically suggest a short duration of the treatment and the prescription of the lowest hormonal dose able to control symptoms, should be followed.

Hormone replacement therapy, based on the current evidence, is not recommended for the prevention of age-related diseases as it carries a high risk/benefit ratio related to:

• increased risk of breast cancer which is directly associated with the duration of the treatment and possibly with the type of estro-progestinic agent used [8];

• RCTs have not demonstrated the effectiveness of HRT for the prevention of cardiovascular diseases, particularly for myocardial infarction, whereas there is evidence for an age-independent increase of the incidence of stroke and deep vein thrombosis (DVT) [9];

• with reference to osteoporotic fractures, a treatment is usually not recommended for their prevention, even if started several decades before the age they become more frequent [10];

• the current evidence suggests that HRT may not have a protective effect on the cognitive decline and on the prevention of dementia.

Natural progesterone-like agents are associated with a lower incidence of breast cancer.

Which alternatives to HRT, especially non-pharmacological, need more information?

It is important that every woman is informed about the availability of pharmacological (HRT) and non-pharmacological treatments.

Menopause could provide the chance to health professionals to recommend changes in lifestyle, which would certainly have beneficial effects towards menopausal symptoms. Two important lifestyle changes to be recommended are the adoption of a healthy and balanced diet and the increase in physical activity in order to reduce the risk of osteoporosis, CVDs, obesity, urinary incontinence and vasomotor symptoms.

The choice of the different non-pharmacological treatments should be supported by educational and counselling sessions on the subject of menopause.

### Priorities for future research

What are the priorities for research?

The jury identified several areas of uncertainties related to HRT which could be investigated in RCTs. However, the feasibility (samples size, resources, ethics) and the clinical relevance of the results should be evaluated before designing any new study. The most significant topic, related to the information and therapeutic demands of women, is the assessment of potential treatments able to control menopausal symptoms and, in general, to improve the quality of life. These studies may require smaller human and financial resources (smaller samples size) and could potentially provide useful clinical information to improve women's quality of life during the menopause.

## Discussion

The approach we used was effective in involving lay people in the debate about HRT and the management of menopause. Particularly, the participation of consumers and journalists in the two specific working groups gave the jury the opportunity to be exposed to a wide range of views through the documents produced. The presence of consumers representatives and journalists in the jury reinforced this contribution: their involvement was particularly significant considering the focus of the CC on the information.

The jury stressed that menopause is not a disease but a phase of life and, as such, does not require medical treatment in itself. This seems in accordance with the attitude of most of the women interviewed in the population survey carried out for this CC: more than 90% of the sample believes that menopause is a normal phase in women's life and almost half of the sample states it is a positive experience for a woman. More than half of the sample did not receive any information about menopause and possible remedies. Lack of knowledge was associated with low educational level and absent or scarce access to health services and prevention opportunities, like female cancer screening. It has been demonstrated that women's attitudes towards the menopause and their knowledge of the benefits and risks of HRT have a direct effect on their use of the therapy [11] and also that clinicians have a strong influence on women's behavior towards HRT [11,12].

Other sources of information are the mass media, that conveys to women messages often low quality and conflicting. This is true both for most of the documents available online, and for many of the articles published in lay press. Some studies have found that women, not trusting in the first opinion given by a medical expert, often have used web based health services to seek a second opinion about a problem related to menopause. This behaviour has almost certainly been adopted by Italian women too, nevertheless, the examination of the information available on internet found that only about 20% of the published material may be useful to make an informed decision about treatments. The analysis suggests that the information about menopause is often poor and not independent. The lack of references of information in almost half of the documents considered is a sign of scarce transparency and does not allow the assessment of the reliability of the content. More and better information should be given to middle-aged women to improve their skill to make informed choices regarding the use of HRT. To reach this goal, the jury stressed the need to improve scientific journalism in Italy. The following aspects should be the priorities for such effort: a better and focused opportunity for training; a better understanding of the complexities in the interpretation of scientific issues and a more critical approach against experts' views and statements. The potential negative influence of conflicts of interests in the validity and reliability of the information should be appreciated; more attention is needed to avoid an unbalanced use of the information as propaganda. The availability of reliable documents produced by institutional sources, with careful selection and description of the references, could be helpful as reference to compare the other sources of information. Similarly to what is already suggested in the professional area, the general public should use appropriate criteria to search for information on internet in order to verify the quality and the independence of the sources.

The jury recommends to prescribe HRT to women with an early onset of the menopause and to women with vasomotor problems and disturbed sleep perceived as important and persistent, not for prevention purposes. It suggests a short duration of the treatment and the prescription of the lowest dose able to control symptoms, as indicated in the approved clinical guidelines. Areas of uncertainties which could be investigated in properly designed studies are identified by the jury. The most significant topic is the assessment of potential treatments able to control menopausal symptoms and to improve quality of life. The analysis of the cost-effectiveness of HRT's effects and observational epidemiological studies, including assessment of behaviour, lifestyle, drugs and alternative therapies, should also be promoted. The most important RCTs have investigated conjugated estrogens, which are scarcely available in Italy and Europe. Most of the results from these studies cannot be generalised because of the age and physical characteristics of women enrolled in these trials (initial phase of menopause) and supposed to take HRT. It would be appropriate to plan studies to investigate the risks associated with the treatments normally used in our daily practice and in women routinely taking them to test their effectiveness and efficacy. This research plan would require large financial and human investments but it could potentially provide important information on the safety (DVT, breast cancer), which has not yet been investigated in other RCTs.

This experience has some limitations due to the methodology of the consensus conferences and to the process required for the preparatory activities. The jury and the three working groups were all appointed by the organising committee according to criteria shared with the scientific board. The working group on clinical issues did not undertake a systematic review of the scientific literature on HRT but appraised the guidelines – published by institutions and scientific societies – and systematic reviews already available and some original articles considered relevant by the group.

The OC disseminated the CC's final statement to reach a wide target and increase the implementation of the results. With this aim, the final recommendations of the Jury have been briefly reported in the Bollettino d'informazione sui farmaci (BIF), the official journal of AIFA, the Italian medicine agency [13].

The dissemination of the CC recommendations and their impact will be evaluated through a project funded by a grant of the Italian Ministry of Health. It will be carried out in several Italian Regions in the next two years in order to support women's decision making process about HRT, and to evaluate the impact of the dissemination activities on HRT prescriptions through a before- after research.

## Conclusion

The consensus conference provides indications about the priorities for information on hormone replacement therapy.

The final document supports the use of hormone replacement therapy in women at the beginning of menopause and suffering for hard-to-bear symptoms or in early menopause; hormone replacement therapy should be used for the shortest possible period and at the lowest effective doses.

The consensus conference's final document recommends that the term "post menopausal hormone therapy" substitutes hormone replacement therapy, in order to avoid that menopause is classified as a syndrome due to lack of hormones.

## Competing interests

The authors declare that they have no competing interests.

## Authors' contributions

The authors actively participated to the planning and conducting of all the phases of the consensus conference described in the article. The working groups and the information surveys were coordinated by PM and CC. The population survey was coordinated by SD. PM and CC drafted the article, and all the authors reviewed it, contributed to the discussion, and gave final approval of the version to be published.

## Appendix 1

* MEMBERS OF THE CONSENSUS CONFERENCE GROUP

### Scientific board

*Alberto Donzelli*, ASL Città di Milano; *Maria Font*, Dipartimento Farmaceutico ULSS 20 di Verona; *Brunello Gorini*, Federazione Italiana Medici di Famiglia (FIMMG) Veneto, Treviso; *Luisa Ronchi*, Movimento Consumatori, ASL Città di Milano; *Carlo Schweiger*, Cardiologo, Milano; *Ludovica Tagliabue*, Altroconsumo Associazione, Milano; *Paolo Zola*, Cattedra di Ginecologia Oncologica, Dipartimento di Discipline Ginecologiche e Ostetriche, Università degli Studi, Torino

### Jury

*Angelo Benessia *(Presidente), Avvocato, Torino; *Luisella Battaglia*, Comitato Nazionale Bioetica, Genova; *Cesare Cislaghi*, Agenzia Nazionale per i Servizi Sanitari Regionali, Roma; *Maria Corongiu*, Federazione Italiana Medici di Famiglia-Lazio, Roma; *Monica Daghio*, Laboratorio Cittadino Competente, AUSL Modena; *Nicola Magrini*, CeVEAS Centro Valutazione Efficacia Assistenza Sanitaria, Modena; *Mariapiera Mano*, Dipartimento Scienze Biomediche Università degli Studi, Torino; *Daniela Minerva*, L'Espresso, Roma; *Rossella Miracapillo*, Movimento Consumatori, Osservatorio Farmaci e Salute, Roma; *Manuela Molinari*, Consultori Familiari ASL Provincia di Mantova; *Rossella Panarese*, Radio 3 Scienza, Roma; *Amedeo Santosuosso*, Corte di Appello, Milano; *Sara Stefania Tabbone*, Associazione Italiana Donne Medico AIDM, Treviso; *Massimo Tombesi*, CSeRMEG Centro Studi e Ricerche in Medicina Generale, Macerata

### Working group "Information and media"

*Maria Grazia Buratti*, Istituto di Ricerche Farmacologiche Mario Negri, Milano; *Daniela Condorelli*, D di Repubblica, Gruppo l'Espresso, Milano; *Cristina D'Amico*, Corriere Salute, RCS Quotidiani, Milano; *Gianna Milano*, Panorama, Mondadori Editore, Segrate (Mi); *Edoardo Rosati*, Oggi, RCS Periodici, Milano; *Daniela Toderini*, Endocrinologo, medico di medicina generale, Padova; *Antonella Trentin*, Donna Moderna, Mondadori Editore, Roma

### Working group "Information and booklet, brochure targeted women"

*Cinzia Colombo*, Istituto di Ricerche Farmacologiche Mario Negri, Milano; *Gloria De Bernardo*, Comitato Etico Azienda Ospedaliera, Verona; *Livia Giordano*, Epidemiologia dei Tumori, Centro Prevenzione Oncologica Piemonte, Azienda Ospedaliera San Giovanni Battista, Torino; *Giovanna Menicatti***, **APM Parkinson Lombardia, Milano; *Rosita Orlandi***, **Federazione Italiana Associazioni Donatori Sangue, Puglia, Comitato Etico Ospedale Consorziale Policlinico, Bari; *Ludovica Tagliabue*, Altroconsumo Associazione, Milano

### Working group "Clinicians"

*Marina Bosisio*, CSeRMEG Centro Studi e Ricerche in Medicina Generale, Monza (Mi); *Emilio Maestri*, Gruppo Area Farmaci e Area Linee Guida, CeVEAS Centro Valutazione Efficacia Assistenza Sanitaria, Modena; *Raffaella Michieli*, Segretario nazionale e responsabile dell'Area Salute della donna della Società Italiana di Medicina Generale, Mestre (Ve); *Fabio Parazzini*, II Clinica Ostetrica Ginecologica, Università degli Studi, Milano; *Carlo Schweiger, C*ardiologo, Milano; *Paolo Zola*, Cattedra di Ginecologia Oncologica, Dipartimento Discipline Ginecologiche e Ostetriche, Università degli Studi, Torino

## Pre-publication history

The pre-publication history for this paper can be accessed here:



## Supplementary Material

Additional file 1 ***Final report *Consensus Conference: Informing women about hormone replacement therapy (HRT)**. This is the final statement written and approved by the Jury of the consensus conference, and officially presented at the end of the celebration of the consensus conference. Click here for file
