# Supplementary material for: Informing women about hormone replacement therapy: the consensus conference statement
Source: BMC Womens Health. 2009 May 29;9:14. doi: 10.1186/1472-6874-9-14 (PMC2698849; doi:10.1186/1472-6874-9-14)
Supplement: Additional file 1 — Final report Consensus Conference: Informing women about hormone replacement therapy (HRT). This is the final statement written and approved by the Jury of the consensus conference, and officially presented at the end of the celebration of the consensus conference. [file 1472-6874-9-14-S1.pdf]

Consensus Conference

Informing women about hormone replacement therapy (HRT)

Final report

1. Introduction

1. The final version of this report was produced at the end of the second workshop as a result of the collaborative effort of the different members of the scientific committee of the Consensus Conference.
2. Like the preliminary version of the report (available online at www.partecipasalute.it), the final version has been written based on the assumptions that all the information, references and data provided by the working groups and by each member of the committee, before and during the conference, are accurate, precise and independent. The committee has not carried out any control on the reliability of the data and therefore cannot be considered liable for any misuse of the data or references used for the preparation of this report. The report is completely independent and impartial. However, the committee has emphasised that this report is not intended to provide clinical guidelines for the prescription of HRT in the general population. The content and reliability of each comment and statement should be professionally and independently assessed because, as clarified in the protocol of the conference (see www.partecipasalute.it), the approach used during the conference and during the preparation of the report may have some methodological flaws.
3. Hence, we advice medical doctors and health professionals to take into account the committee's guidelines published on the website www.partecipasalute.it, and to make an independent analysis of the information before using the report in the clinical practice.

2. The questions

The committee made every reasonable effort to answer to these primary questions according to the targets identified in the original protocol:

1. Which characteristics of the menopause can be circulated as health problems?
2. Why should we prescribe hormonal replacement therapy (HRT), which are the main objectives of the therapy, which women are eligible to receive it and what would be the duration of the treatment?
3. Which alternatives to HRT, especially non-pharmacological, need more information about:
    - Menopausal Symptoms
    - Cardiovascular Diseases (CVDs) prevention
    - Bone Fractures prevention
    - Cancer prevention
4. Which research areas should be targeted in the future?

5. What do women know about menopause and what would they like to know?
6. How to critically interpret the data available on this topic? Which are the main risks associated with the dissemination of deceitful and unreliable information which may influence the general public and professionals? Can the scientific quality of the information be improved?

3. Conclusions

An observation from the committee

This report has been specifically produced for the general public, medical and non-medical experts interested in or working in the area of menopause and HRT. Therefore, we have used two different approaches in order to communicate the main messages: 1) a concise writing style to improve the clarity and dissemination of the information; 2) a specialist and scientific style (in squares) more accessible to medical professionals. The committee is of the opinion that either the full or the short version of the report may be used (acknowledging the availability in the full document of the "motivations" of the recommendations written in the short version). The committee also encourages a clear identification and separation of the recommendations briefly reported from the motivations, more difficult to understand for non-medical readers, in order to make the full document readable using a two-step approach.

The committee also considers the term "hormonal replacement therapy" (HRT) as misleading since it may imply a situation of hormonal deficiency and, consequently, a diseased state. Since this concept is not in line with the one being used in this report, the committee recommends instead the term post-menopausal hormonal therapy (PHT) as more appropriate. However, the term HRT has been chosen in the report and the terminology has not been amended since the document answers to questions formulated by the promoting committee of the Consensus Conference and defined with specific terms, that are consequently not modifiable.

1. Which characteristics of the menopause can be linked to health problems?

Menopause is one of physiological phases in women's life, which could sometimes affect their quality of life.
The medical problems with a proved association with menopause are:
- Vasomotor symptoms (sweating, flushing)
- Vaginal dryness
- Disturbed sleep

Vasomotor symptoms and disturbed sleep are generally short-lasting and variable in their intensity. However, they may significantly affect women's quality of life. Other medical conditions, frequently associated with MS (irritability, depression, osteoarticular pain, weight gain), do not have a causal link with menopause but they would similarly require medical attention.

The only clinical signs and symptoms caused by menopause, proposed by a panel of experts, are vasomotor disturbances, vaginal dryness and related dyspareunia (painful intercourse) and probably disturbed sleep (especially

when associated with night sweating) (1). The proof of a cause-effect relationship between psychological or somatic health problems, such as muskulo-skeletal pain, joint problems, back pain, anxiety, depression and sexual dysfunction not related to vaginal dryness, with the hormonal changes occurring during menopause is missing and for some of them psychological justifications are plausible. However, these health problems can require medical attention, independently of their causal relationship with menopause. Based on this evidence, a direct and beneficial effect of HRT on these symptoms and on the overall quality of life of women beyond the expected effects of HRT on vasomotor and vaginal problems should not be anticipated.

2. Why should we prescribe hormonal replacement therapy (HRT), which are the main objectives of the therapy, which women are eligible to receive it and what would be the duration of the treatment?

Hormonal replacement therapy should be prescribed to women with an early onset of the menopause, considered as a pathological state, and to women with vasomotor problems and disturbed sleep perceived as important and persistent. Vaginal dryness and difficult sexual intercourse (dyspareunia) are not considered criteria for the recommendation of systemic HRT and they can be treated with topical treatments, which are generally effective.

However, women with milder symptoms may have a negative perception of the menopause and therefore they may explicitly request HRT. The criteria for the prescription of HRT in these cases cannot be defined and generalised and the therapeutic plan for each patient should be individually discussed with a medical doctor.

Women should be informed that menopausal symptoms may be short-lasting (with the exception of vaginal dryness) and benign and they should be aware of the risks and benefits associated with HRT, of the frequent recurrence of the symptoms after its interruption and of the availability of non-pharmacological treatments able to alleviate symptoms in order to make an informed and conscious decision.

Every woman should receive updated and clear information on the available non-pharmacological treatments and lifestyle modifications programmes (see Point 3).

Clear evidence about the optimal duration of the treatment to effectively control the symptoms is currently not available. The approved clinical guidelines should be followed, which specifically suggest a short duration of the treatment and the prescription of the lowest hormonal dose able to control symptoms.

Hormonal replacement treatment in premature menopause (< 45years) has not been investigated in randomised clinical trials (RCTs) to explore the long-term effects and the risk-benefit ratio. The current evidence proposes the prescription of HRT in these cases but no precise indication on the duration of the treatment is provided.

In general, the frequency and the intensity of the vasomotor symptoms can be influenced by clinical criteria, ethnicity and socioeconomic status (2). The effects of HRT on menopausal symptoms (flushing with sweating and vaginal dryness, that can be controlled with topical treatments) are well known (3,4). The effects of HRT on the quality of sleep are less documented but it may be possible a beneficial effect in women with intense nocturnal vasomotor symptoms. The current recommendations therefore suggest that, in absence of clinical contraindications (increased cardiovascular and thrombotic risk, high risk or suspect of breast cancer, vaginal bleeding, liver diseases), HRT should be started in women with severe or intolerable vasomotor problems at low doses and for short periods.

The duration of the treatment should be individually tailored based on the periodical reassessment of the clinical problems and on the compliance of the patients, since there are no controlled studies providing specific guidelines on the duration of HRT. However, the following factors may have to be considered:

- Symptoms may have a variable intensity and may be transient (with the exception of vaginal symptoms) in most women; they may disappear after few months in 30-50% of cases and after 4-5 years in 85-90% of women (5).

- A high placebo effect, probably related to the temporary nature of the symptoms. Placebo may reduce symptoms by about 50% compared to treatments with estrogens (85-90%), using different doses and preparations (oral, topical) (6).
- A high incidence of relapse (up to 50%) (7) after the interruption of the treatment or even the occurrence of new symptoms not present at the beginning (8), and therefore the necessity to treat the symptoms is often just delayed.
- The side effects associated with conjugated HRT (mammary tension and vaginal discharges) and the consequent, important increase in diagnostic procedures and performance of hysterectomies (9).

Several women (about 50%) during their perimenopausal transition require medical attention for health problems not related to vasomotor symptoms such as vaginal dryness, insomnia and urinary symptoms. The last ones are not really resolved by the prescription of HRT (10).
The urinary problems associated with abnormal bladder control during menopause have been reviewed and the results published in the Cochrane database. The review included 28 RCTs (including less than 3000 women) and it was published before the publication of the Women Health Initiative Study (WHI) (11). The review concluded that HRT might be more useful for the treatment of incontinence (more for the urge incontinence than for the effort incontinence) (12). The WHI study suggested instead that estrogens alone or in combination with progesterone did not prevent urinary incontinence (either forms or mixed forms) and HRT may increase the severity of the symptoms within a year (13). The risk was increased in women with effort incontinence or treated with estrogens alone. The data do not allow for an analysis stratified by age and there are no plausible reasons to hypothesize an interaction of the treatment with age.

Hormone replacement therapy, based on the current evidence, is not recommended for the prevention of menopausal symptoms as it carries a high risk/benefit ratio related to:

- Increased risk of breast cancer which is directly associated with the duration of the treatment and possibly with the type of estro-progestinic agent being used.
- RCTs have not demonstrated the effectiveness of HRT for the prevention of cardiovascular diseases, particularly for myocardial infarction, whereas there is evidence for an age-independent increase in the incidence of stroke and deep vein thrombosis (DVT).
- With reference to osteoporotic fractures, a treatment is usually not recommended for their prevention even if started several decades before the age they become more frequent. .
- The current evidence suggests that HRT may not have a protective effect on cognitive decline and on the prevention of dementia.

Natural progesterone-like agents are associated with a lower incidence of breast cancer.

Breast cancer

Epidemiological and clinical studies have found a direct association between HRT and breast cancer. The main findings of these studies are (14):

- An increased risk of breast cancer with the duration of the estroprogestinic treatment. The increase in risk could be observed during the first year, it was statistically significant after 5 years and it returned to baseline after 5 years from the discontinuation of the treatment.
- Non-conjugated estrogens carried fewer risks than conjugated estrogens.
- Preparations with micronized progesteron carried fewer risks than synthetic progesterone (15).
- A greater risk was observed in cross sectional studies compared to RCTs (both with conjugated and non-conjugated estrogens)
- Definite data on the effects of estrogen or progestinic preparations or on the combined or sequential administration of the drugs are not available.

RCTs have usually short follow-up periods and they use an intention to treat analysis (ITT), two factors which tend to decrease the ability to detect the effects of the treatment. The data published from the WHI study, taking into account the study duration, did not show a difference in the mortality rate for breast cancer which would require longer follow up periods due to the long latency of onset of cancer.
In addition,

- The WHI study showed that subjects randomised to receive estro-progestinics had an increased incidence of breast cancer during the first 5 years of follow-up (16), a result usually not detected in observational studies.
- Consistent methodological doubts have been related to the lack of correlation between conjugated HRT and risk of breast cancer in treatments with a short duration, linked to the evident exclusion from the studies of women with a recent diagnosis of breast cancer (17). More specifically, women taking HRT and receiving a diagnosis of breast

cancer shortly after the start of the treatment were less likely to be included, which may have determined a systematic bias.

- The publication of the results from the WHI study determined a reduction in the number of HRT prescriptions in the USA, which was followed by a consequent decrease by 6.7% (only in women above 50 years) in the incidence of breast cancer (18). A similar trend has been observed in Canada (19), France (20), Germany (21) and Australia (22). These observations do not prove a causal link between the two phenomena; however, to date, alternative explanations are completely missing (23).

Under these circumstances, the absent risk for breast cancer related to HRT with estro-progestinics taken for a period less than 5 years cannot be confirmed. In addition, the limits of sensitivity and specificity of the main diagnostic procedures (mammography) have to be taken into account to estimate cancer risk in women taking HRT with estro-progestinics. This aspect is particularly relevant for the diagnosis of breast cancer in young women as the tissue of the mammary glands is denser. These histopathological characteristics may delay the detection of early cancerous lesions which may be characterised by a more aggressive stage, which would have important consequences for the prognosis (16, 24). Hormone replacement therapy is associated with an increased performance of invasive diagnostic procedures and false positive mammographic reports.

Decrease in colon cancer

The WHI study (analysis based on subjects randomized to the conjugated therapy) observed a decrease in the incidence of colon cancer (prevention of 6 cases per 10000 women/year) (25), which is in line with the results obtained from observational studies. However, the mortality rate of women taking HRT did not decrease and their prognosis was even more severe due to the more aggressive histo-pathological features of colon cancer, reflecting, most likely, a delayed diagnosis. This may suggest that rectal bleeding, one of the most important sign for the early detection of colon cancer, may be confused with vaginal bleeding, a common effect of HRT, which may defer the identification of the tumour (25).

The decrease in colon cancer incidence cannot be used as an indication for the prescription of HRT but it could be theoretically included in the overall assessment of the risk/benefit ratio associated to the treatment. In women younger than 60 years, the benefits are basically nonexistent since the incidence of colon cancer in this population is low and women with an increased risk (familiar polyposis) to develop colon cancer have frequent and specific tests (endoscopy) to detect lesions as early as possible.

Cardiovascular risk and stroke

The timing hypothesis proposes an interaction between HRT and the biological age of women (26). This hypothesis stems from the contradictory results of observational studies (positive association) and RCTs (negative association). The hypothesis proposes that estrogens may prevent the formation of atherosclerotic plaques but they may have damaging effects on the existing plaques. However, observational studies are characterised by a significant selection bias and a recent meta-analysis (WHI study was not included) failed to demonstrate any beneficial effect of HRT on CVD risk (27).

The negative results on CVD risk from the WHI study, published in 2002, did not show any interaction with age. The study observed an increase in ischaemic heart disease, especially during the first year of treatment, and the results were more convincing (caution should be used in the interpretation) when the analysis included subjects completing the trial (i.e., results were not based on an ITT analysis). The risk for ischaemic heart disease remained unchanged in women with hysterectomy under treatment with non-conjugated estrogens and an excess number of ictus remains. The positive trend observed in women between 50-59 years was not statistically significant (28). The results from the WHI study looking at the frequency of events, stratified by age, were published the following year (29). The analysis showed the same negative results when age or number of years since the onset of menopause were used, confirming the lack of interaction with age. The analysis by age groups (50-59, 60-69, 70-79 years), carried out in the WHI study, supported neither a protective effect nor a reduced risk associated with HRT in younger women.

Subsequently the results were reanalysed using different and more complex statistical methods aiming at improving the power of the study to verify the consistency of the timing hypothesis (30). The authors clarified that this analysis was not originally planned and that the nature of the analysis was entirely speculative to test the consistency of the timing hypothesis. First, the data were pooled together which probably influenced negatively the generalizability of the results to the clinical practice, since HRT is mandatory in women without hysterectomy. The analysis showed more clearly that the positive trends of ischaemic heart disease and mortality rate were stronger but still not significant. Similarly, the ictus trend (which had a tendency to increase after about 1.5 years from the start of the estro-progestinic therapy) was not in agreement with the timing hypothesis, even when age (below 60 years) or menopausal age were taken into account (31). Finally, the pooled data showed that the risk of stroke in women younger than 60 years or during the first 10 years of menopause was not reduced.

More recently, the WHI-CACS (32) study observed a decrease in coronary calcifications (an indicator of atherosclerosis) in a subgroup of women enrolled in the WHI study (women with hysterectomy and treated with estrogens alone). The results, limited to women between 50-59 years, showed a substantial reduction of coronary calcifications in women treated with estrogens compared to placebo. The outcome variable is a proxy measure and the duration of the effects on the reduction of coronary calcifications was unknown. In addition, it was not clear whether a

reduction in coronary calcifications was associated with a decrease in cardiovascular events in women at higher risk for CVD because of their age (during 7 years of treatment no changes were observed). The predictive value of coronary calcifications in women carrying a low risk, like 50-60 years old women eligible for HRT, was poor and the same authors did not recommend HRT for the prevention of CVDs (33, 34).

The timing hypothesis is an interesting concept, not supported by clinical outcomes of RCTs, which showed neither a significant reduction in CVD events in women treated with HRT before 60 years nor an interaction of the estrogenic effects with age (35). Several other analyses, based on the WHI data, have tested the validity of the timing hypothesis and they have unanimously concluded that HRT (with or without progestinics) is clinically beneficial for the short-term treatment of severe and not tolerated menopausal symptoms. The same studies concluded that new and well-designed studies are necessary to test the hypothesis. Peri-menopausal women without a history of cardiovascular risk factors and without clinical evidence of atherosclerotic disease have a low absolute cardiovascular risk (and a pharmacological treatment would not be required in these cases) (32, 36). None of the main clinical guidelines for the prescription of HRT or for the management of cardiovascular risk factors recommends HRT for the prevention of CVDs (7, 37, 38, 39).

Venous thrombo-embolism

The WHI study showed an increased risk for deep venous thrombosis (DVT) and pulmonary embolism with oral conjugated estrogens and progestinics (12 excess cases every 10000 years/woman) independent of age - including women between 50-59 years old - whereas the treatment with estrogen only induced a tendency towards higher risk for DVT and pulmonary embolism (2 excess cases every 10000 years/woman) not statistically significant (40). The risk is higher after the start of the therapy and returned to baseline after stopping the treatment. The risk increased with age and overweight but there was not an identifiable association with other cardiovascular risk factors, which could be used as screening for women at increased risk and consequently prevent them from having the treatment. Even if an increase in incidence was observed in women carrying the V Leiden factor, most of the cases occurred in women without thrombophilic disorders. The screening for thrombophilic disorders is therefore not useful in subjects without a history of DVT, which would be a contraindication to HRT in any case. The risk in young women, very low in absolute terms, was nearly doubled by the estroprogestinic therapy according to an ITT analysis and it was directly associated with compliance to HRT.

A recent meta-analysis has observed that the risk is significantly reduced by the use of transdermic estroprogestinic compared to oral preparations (41). The data on the transdermic preparations are observational but they may have a biological plausibility as the prothrombotic action of the estrogens is mainly determined by hepatic metabolism, which is not activated by the transdermic administration of the drugs. The meta-analysis did not observe differences between estrogens and estroprogestinics, a result which is in contrast with the WHI study.

A multicenter case-control study has showed that different classes of progestinics may have a different risk for DVT. A greater risk (RR 3,9; CI95% 1,5-10) was associated with nor-pregnan derived agents whereas micronized progesterone and pregnan derived molecules did not increase the risk for DVT (42).

Bone fractures

HRT with estrogens alone or in combination determines an increase in spine and hip bone mineral density (BMD) and a significant reduction in bone fractures. The WHI study showed a 24% decrease in the total number of fractures (-34% hip fractures which is nearly equal to 1 fracture prevented per year/per 2000 women; reduction of forearm and vertebral fractures by 29% and 35%, respectively) (43). Most of the participants in the WHI study were not osteoporotic. The decrease in the absolute risk was about 2.5% (NNT= 40 at 5.6 years) for the occurrence of any fracture in a population with an average age of 63 years. However, the authors concluded that HRT was not beneficial not even in women at high risk of fractures and the conclusion was also true for women with hysterectomy (44).

The increase in BMD with estrogen therapy is well documented but there is no additional gain in BMD after 2-3 years of therapy, even if not necessarily there is a rapid decrease in BMD after the suspension of HRT (45). The data on the duration of the protective effects on bone health after the suspension of HRT are inconsistent. A small study (347 women) indicated a prolonged effect of HRT (46), whereas another study (Million Women Study) suggested that the beneficial effects of estrogens rapidly disappeared after discontinuing the treatment and after only one year the incidence of fractures of women taking HRT was similar to women not taking HRT (controls) (47).

The risk of hip and vertebral fractures is directly associated with age and it becomes very important after 65-70 years; hence the effects of HRT on the risk for severe fractures (femur, spine) between 50 and 60 years are in absolute terms negligible. Osteoporotic fractures are an important problem in older women and the hypothesis to prevent them with pharmacological therapies like HRT started 15-20 years in advance and interrupted 10-15 years before the significant increase in fracture risk, does not seem applicable in women carrying a normal risk for fractures. For cases with a particularly high risk, like for example in a situation of secondary prevention, there is a large availability of other effective drugs. Therefore, HRT should only be prescribed to the selected group of women carrying a high absolute risk of fractures (secondary prevention, secondary osteoporosis) when other pharmacological agents cannot be used.

Dementia and mild cognitive impairment

The neurological effects of estrogens on psychological status, depression, cognitive functions and risk for degenerative diseases like Parkinson and Alzheimer diseases are important research topics. A neurological timing hypothesis has been proposed to suggest the possible beneficial effects of an early start of HRT on neurological disorders (48). However, a clear therapeutic window has not yet been identified and screening criteria for the identification of women at increased risk are not yet available. The WHI-MS study in women above 65 years old observed that HRT with estroprogestinics increased the risk of dementia (23 more cases/10000 women/year), did not prevent mild cognitive decline and did not improve cognitive function (49-50). There are no RCTs exploring the influence of HRT on the long term course of these diseases in young women. A 3-year cohort study in USA (UTAH), in women with an average age of 73 years, analysed retrospective data on HRT (51). The study found a decrease in dementia in women previously treated with HRT. A case control study (including contraceptive use after 35 years) supported this hypothesis for the prevention of early dementia (52). Conversely, the Framingham study did not report protective effects on cognitive function (53). Confounding factors have to be taken into account for the interpretation of the results in observational studies, such as the healthy user bias and the recall bias by women with a recent diagnosis of dementia. To summarize, there are no data establishing a protective effect of HRT on the risk of dementia or cognitive decline (54).

3. Which alternatives to HRT, especially non-pharmacological, need more information

It is important that every woman is informed about the transient and benign nature of menopausal symptoms as well as about the availability of pharmacological (HRT) and non-pharmacological treatments.

Menopause could also provide the chance to health professionals to recommend changes in lifestyle, which would certainly have beneficial effects beyond menopausal symptoms. Two important lifestyle changes to recommend are the increase in physical activity and the adoption of a healthy and balanced diet in order to reduce the risk of osteoporosis, CVDs, obesity, urinary incontinence and vasomotor symptoms.

The choice of the different non-pharmacological treatments should be supported by educational and counselling sessions on the subject of menopause.

Diet during the menopause

It is important to remember that:

- During the menopausal phase the energy and nutrients requirements, due to the characteristic hormonal fluctuations, may change considerably to an extent that often energy intake, particularly food with a high energy density, has to be adjusted in order to prevent weight gain.
- Some data suggest that natural estrogens from soy, soy extracts and several other natural sources could alleviate menopausal symptoms. Asian women seem to have less vasomotor symptoms (55).
- Adequate changes in dietary habits could be beneficial to prevent other diseases associated with aging such as coronary heart disease (56, 57, 58), cancer (breast, colon) (59) and osteoporosis (60, 61, 62).

Several other herbal products advertised for the relief of menopausal symptoms have been studied (Cimicifuga, red trifoglio, Dong quai o Chinese herb, Enotera oil) and the results have been often negative (63). Cimifuga racemosa seems to reduce flushing but clinical studies have not tested its effectiveness after 6 months and the long-term safety (64).

Physical activity and menopause

The effect more frequently investigated is associated with the reduction of cardiovascular morbidity and mortality (65, 66). However, the positive effects of physical activity (even with surrogate outcomes) have been demonstrated in post-menopausal women especially for the prevention and treatment of hypertension (67, 68), dyslipidaemia (69), muskulo-skeletal problems (70), mood and overall quality of life (71, 72) (including sleep quality). Physical activity reduces estrogens levels, increases levels of Sex Hormone Binding Globulin (SHBG) (73, 74), and consequently is able to reduce vasomotor symptoms and it has a strong positive interaction with dietary habits (75). A review by the World Cancer Research Fund (76) suggests a positive effect of physical activity on the risk of breast (77) and colon cancer (78, 79). Protective and beneficial effects can be achieved with minor increases in physical activity levels (80) and the current recommendations encourage at least 30 minutes of moderate physical activity (walking) 5 days a week.

4. Where to concentrate the research initiatives?

The committee has identified several areas of uncertainties related to menopause which could be investigated in RCTs. However, the feasibility (samples size, resources, ethics) and the clinical relevance of the results should be evaluated before designing any new study. The most significant topic, related to the information and therapeutic demands of women, is the assessment of potential treatments able to control menopausal symptoms and, in general, to improve the quality of life. These studies may require smaller human and financial resources (smaller samples size) and could potentially provide useful clinical information to improve women's quality of life during the menopause.

Advanced analytical methods should be used to interpret the results and produce more detailed outcome measures. The contradictory results observed so far (and therefore their weakness) are mostly related to the qualitative nature of the main outcomes (quality of life), which makes difficult any robust statistical analysis. The analysis of the cost-effectiveness of HRT's effects and observational epidemiological studies, including assessment of behaviour, lifestyle, drugs and alternative therapies, should be promoted.
The most important RCTs have investigated conjugated estrogens, which are scarcely available in Italy and Europe. Most of the results from these studies cannot be generalised because of the age and physical characteristics of women enrolled in these trials (initial phase of menopause) and supposed to take HRT. It would be appropriate to plan studies to investigate the risks associated with the treatments normally used in our daily practice and in women routinely taking them to test their effectiveness and efficacy. This research plan would require large financial and human investments but it could potentially provide important information on the safety (DVT, breast cancer), which has not yet been investigated in other RCTs.

5. What kind of information about menopause and HRT women would be interested in?

The information provided to women and to health operators should always be independent and based on an evidence-based medicine approach in order to:

- Explore the concept of menopause as part of the natural life cycle.
- Identify symptoms related to the menopause, their duration and the available treatments.
- Understand the risk and benefits of the treatments (pharmacological and alternative therapies).
- Incorporate lifestyle changes in any treatment plan.

During the peri-menopausal period it is important that women realize the possibility of procreation as well as that natural contraceptive methods may not be totally effective.

The research carried out by the task force of the Consensus Conference has selected and reviewed 78 documents published online. Twenty-four were published by the national health system (NHS) and by scientific societies and 54 by private sources (pharmaceutical companies, publishing houses and patients associations) (81). A separate analysis investigated 225 articles published in newspapers, magazines and scientific magazines (82).
A closer examination noticed that some articles, particularly if published online, were produced using a non professional style, they were poorly written and had an inadequate graphical quality. Aspects like the frequent updates of the website information, easy access, fast download of the web site content, prominent position on the webpage of the main search engines and high quality presentation did not authenticate the truthfulness of the information (83). Nevertheless, some studies have observed that certain factors mentioned earlier may influence the personal opinion about the validity of the information retrieved online.
The health information was frequently linked (50% of the articles) to commercial activities or sponsors, particularly for herbal products. The scientific sources were often not reported and 20% of the articles were written by an expert in the field (the general tendency of the public is to attribute the articles published online to experts working in health organizations (84). This is not necessarily true and it may give false credibility to the articles).

Some studies conducted abroad have found that women, not trusting the first opinion given by a medical expert, often have used web based health services to seek a second opinion about a problem related to menopause (85, 86). This behaviour has almost certainly been adopted by Italian women too.
A careful examination by the task force of the information available about menopause and/or HRT (websites, leaflets, booklets, fliers) found that only about 20% of the published material may be useful to make an informed decision about treatments. In addition there is a scarcity of information produced by the Italian NHS, by patients associations and by scientific societies (81). The task force concluded that about 50% of the articles published in life magazines and specialized periodicals might be helpful, but only 25% of the articles in women's magazines and 30% in health magazines reported reliable information. The review emphasised the primary role of the NHS to promote a capillary and direct diffusion of independent information able to reach the general public and health operators through the organization of training courses (continuous medical education, CME).
Every woman should be able to exercise her right to take a conscious and informed decision after she has had access to qualified sources of information able to provide definite answers to questions related to menopause, therapies, risks, uncertainties still present in research and possible non-pharmacological treatments.

6. How to critically interpret the information available on this health issue? Which are the main risks for the general public and professionals associated with the dissemination of deceitful and unreliable information? How to improve the scientific quality of the information?

The first articles published about HRT have initially highlighted benefits, subsequently revised by studies published in the medical literature, which have characterised the risks associated with the treatment. The overall quality of the information about menopause (professional and non-professional sources) is poor and it has clear methodological flaws, contradictions and conflicts of interests (economic and professional). Actually, the risk related to this misinformation is that women with non tolerated menopausal symptoms would be prevented from having HRT even though they could benefit from the treatment with relatively modest risks. Therefore, it is critical to deliver scientific and correct information on the current knowledge, advantages and disadvantages, including areas of uncertainties, pharmacological and non-pharmacological alternative treatments and their effectiveness.
The information should neither promote the use of HRT nor raise concern about the use of HRT. Every therapeutic decision should be based on an empathic patient-doctor relationship to take into account the preferences and priorities of every patient.

The information about HRT before the publication of the WHI study was strongly biased by the supposed preventative effects, mainly on the cardiovascular system, without taking into account the several comments on the probable selection bias occurred in most of the non-randomized clinical trials (87, 88). The Italian population was characterised by a smaller CVD risk, compared to cancer risk, than other industrialised countries. Consequently the risk-benefit ratio in Italy would have been unfavourable even taking into account the positive effects of HRT on the cardiovascular risk observed in other observational studies (89, 90). In addition, unverified health effects like the improvement of quality of life (these outcomes could be only investigated in double-blind RCTs) were used to promote the treatment.
After the publication of the WHI study, the supposed beneficial effects of HRT were questioned and the recommendations, not without a lively debate, were based on conclusive results rather than on speculations on the potential positive effects of HRT on various diseases, which should be investigated more precisely in experimental studies and clinical research trials. As a consequence HRT use decreased drastically everywhere. However, it could be speculated that because of the contradictory opinions around this issue, some women may have decided to not use HRT notwithstanding the presence of disturbing menopausal symptoms which may have been resolved by HRT.
The analysis of the data published online suggests that the quality of the data about health is variable and the information about menopause is often poor and not independent (91, 92). In Italy, issues related to conflicts of interest in articles for the general public are generally presented in a limited number of cases, despite the opinion of experts, who comment on and provide practical recommendations about menopause, is the reference source most frequently chosen (2/3 of articles).The information published in specialised journals is based on a more reliable and scientific approach.

The task force examined the material published on websites, leaflets, booklets from public and private resources and the information on sponsorship is missing in most of the cases. In 30-50% of cases the original sources of information were not referenced, which was a sign of scarce transparency and does not allow the assessment of the reliability of the content (81).
Menopause is described as a physiological phase of the life cycle in less than 50% of articles and in about 33% the health problems are presented as a disease, which may potentially raise doubts about the general health of every woman.
Most of the articles published in different journals included pictures of women much younger than women most likely to read the article.
Hormone replacement therapy is mainly suggested for the treatment of symptoms but also to improve the quality of life and mood, even if the effects of HRT are not yet confirmed. If we consider websites material, leaflets, booklets written explicitly for a female readership, HRT is described as a preventative measure in about 75% of the pharmaceutical and specialised documents and in about 50% of the documents produced by the NHS and scientific societies. The main indication to HRT is the prevention of bone fractures but in about 50% of documents published by scientific societies and publishing houses, often not datable ,HRT is recommended for the prevention of CVD events, which is currently not supported by the medical evidence.
The risks associated with HRT (the one mainly reported is breast cancer) are discussed in about 60-80% of the documents published by scientific societies, NHS, publishing houses, patients' associations, and in less than 50% of pharmaceutical and specialised documents (82).
Since the most reliable sources of information, especially on the web, are in English, a better quality of the distributed information implies and goes hand in hand with a general qualitative and quantitative growth of the scientific journalism in Italy. The following aspects should be targeted:

- A better training of scientific and medical journalists.
- Journalists should have a better understanding and a deeper critical approach to the discussion of scientific issues and a more constructive interaction with experts.
- Declaration of conflicts of interests related to the issue reported to increase the quality of the information as an indicator of transparency and to validate the source of information.
- More attention to avoid an unbalanced use of the information as propaganda.
- Production of official reports with careful selection and description of the references. The information should be easily accessible and reliable and it could be used by the general public as reference to compare the other sources of information.

Similarly to what is already established in the professional area, the general public should use appropriate criteria to search for information on internet in order to verify the quality and independence of the sources.

Milan, 25 June 2008
For the jury:
The President:
Angelo Benessia

The members of the jury are*:* *Angelo Benessia* (Presidente), avvocato, Torino; *Luisella Battaglia*, Comitato Nazionale Bioetica, Genova; *Cesare Cislaghi*, Agenzia Nazionale per i Servizi Sanitari Regionali, Roma; *Maria Corongiu*, Federazione Italiana Medici di Famiglia-Lazio, Roma; *Monica Daghio*, Laboratorio Cittadino Competente, Sistema Comunicazione e Marketing sociale, AUSL Modena; *Nicola Magrini*, CeVEAS Centro Valutazione Efficacia Assistenza Sanitaria, Modena; *Mariapiera Mano,* Dipartimento Scienze Biomediche Università degli Studi, Torino**;** *Daniela Minerva,* L'Espresso, Roma; *Rossella Miracapillo*, Movimento Consumatori, Osservatorio Farmaci e Salute, Roma; *Manuela Molinari,* Consultori Familiari ASL Provincia di Mantova, Mantova; *Rossella Panarese*, Radio 3 Scienza, Roma; *Amedeo Santosuosso*, Corte di Appello, Milano; *Sara Stefania Tabbone*, AIDM Associazione Italiana Donne Medico, Treviso; *Massimo Tombesi*, CSeRMEG Centro Studi e Ricerche in Medicina Generale, Macerata.

Promoting committee: PartecipaSalute project (Mario Negri Institute, Italian Cochrane Centre, Zadig), National Guidelines System (Istituto Superiore di Sanità).
The project was realized with the support of the Compagnia di San Paolo.
Further information: costa@marionegri.it.

References

1. NIH State-of-the-Science Panel. National Institutes of Health State-of-the-Science Conference Statement: Management of Menopause-Related Symptoms. Ann Intern Med. 2005;142:1003-13.
2. Avis NE, Stellato R, Crawford S, et al. Is there a menopausal syndrome? Menopausal status and symptoms across racial/ethnic groups. Soc Sci Med. 2001;52:345-56.
3. MacLennan AH, et al. Oral oestrogen and combined oestrogen/progestogen therapy versus placebo for hot flushes. Cochrane Database of Systematic Reviews 2004, Issue 4. Art. No.: CD002978.
4. Suckling JA, et al. Local oestrogen for vaginal atrophy in postmenopausal women. Cochrane Database of Systematic Reviews 2006, Issue 4. Art. No.: CD001500.
5. Kronenberg F. Hot flashes: epidemiology and physiology. Ann NY Acad Sci. 1990;592:52-86, 123-33.
6. Nelson HD. Commonly used types of postmenopausal estrogen for treatment of hot flashes: scientific review. JAMA. 2004;291:1610-20.
7. Estrogen and progestogen use in peri-and postmenopausal women: March 2007 position statement of The North American Menopause Society. Menopause. 2007;14:168-82.
8. Ockene JK, et al. Symptom Experience After Discontinuing Use of Estrogen Plus Progestin. JAMA. 2005;294:183-93.
9. The Women's Health Initiative Investigators. Effects of Estrogen Plus Progestin on Gynecologic Cancers and Associated Diagnostic Procedures: The Women's Health Initiative Randomized Trial. JAMA. 2003;290:1739-48.
10. Connelly M, et al. Patient-identified needs for hormone replacement therapy counseling: a qualitative study. Ann Intern Med. 1999;131:265-8.
11. Writing Group for the WHI Investigators. Risks and benefits of estrogen plus progestin in healthy postmenopausal women: principal results From the Women's Health Initiative randomized controlled trial. JAMA. 2002; 288:321-33.
12. Moehrer B, et al. Oestrogens for urinary incontinence in women. Cochrane Database of Systematic Reviews 2003, Issue 2. Art. No.: CD001405.
13. Hendrix SL, et al. Effects of estrogen with and without progestin on urinary incontinence. JAMA. 2005;293:935-48.
14. Collins JA, et al. Breast cancer risk with postmenopausal hormonal treatment. Human Reproduction Update. 2005;11:545-60.
15. Fournier A, et al. Unequal risks for breast cancer associated with different hormone replacement therapies: Results from the E3N cohort study. Breast Cancer Res Treat. 2008;107: 103-11.
16. Chlebowski RT, et al; for the WHI Investigators. Influence of Estrogen Plus Progestin on Breast Cancer and Mammography in Healthy Postmenopausal Women. The Women's Health Initiative Randomized Trial. JAMA. 2003;289:3243-53.
17. Berrino F. Prevenzione in menopausa: punto e a capo. Epidemiologia e Prevenzione. 2002;26(6):269-72.
18. Ravdin P, et al. The decrease in breast-cancer incidence in 2003 in the United States. N Engl J Med. 2007;356:1670-4.
19. Public Health Agency of Canada. Cancer surveillance on-line. http://dsol-smed.phac-aspc.gc.ca/dsol-smed/cancer/index_e.html (Accesso 4/2/2008).
20. Allemand H, et al. Baisse de l'incidence des cancers du sein en 2005 et 2006 en France : un phénomène paradoxal. Bull Cancer. 2008;95 (1):11-5.
21. Katalinic A, Rawal R. Decline in breast cancer incidence after decrease in utilisation of hormone replacement therapy. Breast Cancer Res Treat. 2008;107(3):427-30.
22. Kanfell K, et al. Decrease in breast cancer incidence following a rapid fall in use of hormone replacement therapy in Australia. Med J Aust. 2008;188:641-4.
23. Ravdin PM, et al. A Decline in Breast-Cancer Incidence: Authors reply (Correspondence). N Engl J Med. 2007;357:513.
24. Rowan T, et al. for the Women's Health Initiative Investigators. Estrogen Plus Progestin and Breast Cancer Detection by Means of Mammography and Breast Biopsy. Arch Intern Med. 2008;168:370-7.
25. The Women's Health Initiative Investigators. Estrogen plus Progestin and Colorectal Cancer in Postmenopausal Women. N Engl J Med. 2004;350:991-1004.
26. Grodstein F, et al. Understanding the Divergent Data on Postmenopausal Hormone Therapy. N Engl J Med. 2003;348:645-50.
27. Humphrey LL, et al. Postmenopausal Hormone Replacement Therapy and the Primary Prevention of Cardiovascular Disease. Ann Intern Med. 2002;137:273-84.
28. Hsia J, et al. for the Women's Health Initiative Investigators. Conjugated Equine Estrogens and Coronary Heart Disease. The Women's Health Initiative. Arch Intern Med. 2006;166:357-65.
29. Manson JE. Estrogen plus Progestin and the Risk of Coronary Heart Disease. N Engl J Med. 2003;349:523-34.
30. Rossouw JE, et al. Postmenopausal Hormone Therapy and Risk of Cardiovascular Disease by Age and Years Since Menopause. JAMA. 2007;297:1465-77.

31. Wassertheil-Smoller S, et al. for The WHI Investigators. Effects of Estrogen plus Progestin on Stroke in Postmenopausal Women. The Women's Health Initiative: a randomised Trial. JAMA. 2003;289:2673-84.
32. Manson JE, et al. for the WHI and WHI-CACS Investigators. Estrogen Therapy and Coronary-Artery Calcification. N Engl J Med. 2007;356:2591-602.
33. Greenland P, et al. Coronary artery calcium score combined with Framingham score for risk prediction in asymptomatic individuals. JAMA. 2004;291:210-5.
34. ACC/AHA 2007 clinical expert consensus document on coronary artery calcium scoring by computed tomography in global cardiovascular risk assessment and in evaluation of patients with chest pain. J Am Coll Cardiol. 2007;49:378-402.
35. Barrett-Connor E. Hormones and Heart Disease in Women: The Timing Hypothesis. Am J Epidemiol. 2007;166:506-10.
36. Manson J, et al. WHI-CACS Authors Reply (Correspondence). N Engl J Med. 2007;357:1254.
37. Institute for Clinical Systems Improvement. Menopause and Hormone Therapy (HT): Collaborative Decision-Making and Management (2006). http://www.icsi.org/ (Accesso 26/6/2008)
38. U.S. Preventive Services Task Force. Hormone Replacement Therapy for the Prevention of Chronic Conditions in Postmenopausal Women (2005). http://www.ahrq.gov/clinic/uspstf/uspspmho.htm (Accesso 26/6/2008)
39. Society of Obstetricians and Gynaecologists of Canada. Canadian Consensus Conference on Menopause, 2006 Update. http://www.sogc.org/guidelines/public/171E-CONS-February2006.pdf (Accesso 26/6/2008)
40. Cushman Met al; for the Women's Health Initiative Investigators. Estrogen Plus Progestin and Risk of Venous Thrombosis. JAMA. 2004;292:1573-80.
41. Canonico M, et al. Hormone replacement therapy and risk of venous thromboembolism in postmenopausal women: systematic review and meta-analysis. BMJ. 2008;336:1227-31.
42. Canonico M, et al. Hormone therapy and venous thromboembolism among postmenopausal women: impact of the route of estrogen administration and progestogens: the ESTHER study. Circulation. 2007; 115:840-5.
43. The Women's Health Initiative Investigators. Effects of Estrogen Plus Progestin on Risk of Fracture and Bone Mineral Density. JAMA. 2003;290:1729-38.
44. Jackson RD, et al. Effects of conjugated equine estrogen on risk of fractures and BMD in postmenopausal women with hysterectomy: results from the women's health initiative randomized trial. J Bone Miner Res. 2006;21:817-28.
45. Greendale GA, et al, for the PEPI Safety Follow-Up Study (PSFS) Investigators. Bone mass response to discontinuation of long-term hormone replacement therapy: results from the Postmenopausal Estrogen/Progestin Interventions (PEPI) Safety Follow-up Study. Arch Intern Med. 2002;162:665-72.
46. Bagger YZ, et al. Two to three years of hormone replacement treatment in healthy women have long-term preventive effects on bone mass and osteoporotic fractures: the PERF study. Bone. 2004;34:728-35.
47. Banks E, et al. for the Million Women Study Collaborators. Fracture Incidence in Relation to the Pattern of Use of Hormone Therapy in Postmenopausal Women. JAMA. 2004;291:2212-20.
48. Morrison JH, et al. Estrogen, Menopause, and the Aging Brain: How Basic Neuroscience Can Inform Hormone Therapy in Women. The Journal of Neuroscience. 2006;26:10332-48.
49. The Women's Health Initiative Memory Study: A Randomized Controlled Trial. Estrogen Plus Progestin and the Incidence of Dementia and Mild Cognitive Impairment in Postmenopausal Women. JAMA. 2003;289:2651-62.
50. The Women's Health Initiative Memory Study: A Randomized Controlled Trial. Effect of Estrogen Plus Progestin on Global Cognitive Function in Postmenopausal Women. JAMA. 2003;289:2663-72.
51. Zandi PP, et al; for the Cache County Memory Study Investigators. Hormone Replacement Therapy and Incidence of Alzheimer Disease in Older Women. The Cache County Study. JAMA. 2002;288:2123-9.
52. Henderson VW, et al; for the MIRAGE Study Group. Postmenopausal hormone therapy and Alzheimer's disease risk: interaction with age. J Neurol Neurosurg Psychiatry. 2005;76:103-5.
53. Seshadri S, et al. Postmenopausal estrogen replacement therapy and the risk of Alzheimer disease. Arch Neurol. 2001;58:435–40.
54. Petitti DB, et al. Prevelence of dementia in users of hormone replacement therapy as defined by prescription data. J Gerontol A Biol Sci Med Sci. 2002;57:M532-M538.
55. Lethaby AE, et al. Phytoestrogens for vasomotor menopausal symptoms. Cochrane Database Syst Rev. 2007 Oct 17;(4).
56. Mosca L, et al. for the Expert Panel/Writing Group. Evidence-Based Guidelines for Cardiovascular Disease Prevention in Women: 2007 Update. JACC. 2007;49:1230–50.
57. Anderson JW et al. Meta-analysis of the effect of soy protein intake on serum lipids. N Engl J Med. 1995;333:276-82.
58. Church TS, et al. Effects of Different Doses of Physical Activity on Cardiorespiratory Fitness Among Sedentary, Overweight or Obese Postmenopausal Women With Elevated Blood Pressure: A Randomized Controlled Trial. JAMA. 2007;297:2081-91.
59. Vainio H, Bianchini F. Weight control and physical activity. Lyon: IARC Handbooks of cancer prevention. Vol 6, 2002.
60. Feskanich D, et al. dietary calcium, and bone fractures in women: a 12-year prospective study. Am J Public Health. 1997;87:992-7.

61. Cumming RG, Klineberg RJ. Case-control study of risk factors for hip fractures in the elderly. Am J Epidemiol. 1994;139:493-503.
62. Huang Z, et al. Nutrition and subsequent hip fracture risk among a national cohort of white women. Am J Epidemiol. 1996;144:124-34.
63. Pinato S, Luppino MR. Fitoestrogeni e menopausa. Dialogo sui farmaci. 2003;n°3:173-5.
64. Kronenberg F, Fugh-Bernnan A. Complementary and Alternative Medicine for Menopausal Symptoms: A Review of Randomized, Controlled Trials. Ann Intern Med. 2002; 137: 805-13.
65. Church TS, et al. European cardiovascular disease statistics 2008, European Heart Network.
66. U.S. Department of Health and Human Services. Physical Activity and Your Heart. NIH Publication No. 06-5714, June 2006.
67. Skinner JS, et al. Effects of Different Doses of Physical Activity on Cardiorespiratory Fitness Among Sedentary, Overweight or Obese Postmenopausal Women With Elevated Blood Pressure: A Randomized Controlled Trial. JAMA. 2007;297:2081-91.
68. Seals DR, et al. Effect of regular aerobic exercise on elevated blood pressure in postmenopausal women. Am J Cardiol. 1997;80:49-55.
69. Stefanick ML, et al. Effects of diet and exercise in men and postmenopausal women with low levels of HDL cholesterol and high levels of LDL cholesterol. N Engl J Med. 1998;339:12-20.
70. Kelley GA. Exercise and regional bone mineral density in postmenopausal women: a meta-analytic review of randomized trials. Am J Phys Med Rehabil. 1998;77:76-87.
71. Sutton GL. Quality of life in obese postmenopausal women. Menopause Int. 2008;14:26-32.
72. Pines A, Berry EM. Exercise in the menopause – an update. Climacteric. 2007;10(Suppl 2):42–6.
73. McTiernan A et al. Relation of BMI and physical activity to sex hormones in postmenopausal women. Obesity (Silver.Spring). 2006;14:1662-77.
74. Tworoger SS, et al. Physical activity and inactivity in relation to sex hormone, prolactin, and insulin-like growth factor concentrations in premenopausal women. Cancer Causes Control. 2007;18:743-52.
75. Thompson JL, et al. Effects of diet and exercise on energy expenditure in postmenopausal women. Am J Clin Nutr. 1997;66:867-73.
76. WCRF/ AICR Food, Nutrition, Physical Activity, and the Prevention of Cancer: a Global Perspective 2007.
77. Bianchini F, et al. Weight control and physical activity in cancer prevention. Obes Rev. 2002;3:5-8.
78. Sanjoaquin MA, et al. Nutrition, lifestyle and colorectal cancer incidence: a prospective investigation of 10998 vegetarians and non-vegetarians in the United Kingdom. Br J Cancer. 2004;90:118-21, 23.
79. Wu AH, et al. Alcohol, physical activity and other risk factors for colorectal cancer: a prospective study. Br J Cancer. 1987;55:687-94.
80. Manson JE, et al. Walking compared with vigorous exercise for the prevention of cardiovascular events in women. N Engl J Med. 2002;347:716-25.
81. Documento del Gruppo di lavoro sull'informazione alla donna della Conferenza di consenso. http://www.partecipasalute.it/cms/files/04_tos_Colombo.pdf (Accesso 01/07/2008)
82. Documento del Gruppo di lavoro sull'informazione dei media della Conferenza di consenso. http://www.partecipasalute.it/cms/files/03_tos_Condorelli.pdf (Accesso 01/07/2008)
83. Eysenbach G, Kohler C. How do consumers search for and appraise health information on the world wide web? Qualitative study using focus groups, usability tests, and in-depth interviews. BMJ. 2002;324:573–6.
84. Cumming GP, Currie H. The Internet and the menopause consultation: menopause management in the third millennium. J Br Menopause Soc. 2005; 11:103-8.
85. Hope S, et al. Survey of British women's views on the menopause and HRT. J Br Menopause Soc. 1998; 44:33–6.
86. Hope S, Rees M. Why do British women start and stop hormone replacement therapy? J Br Menopause Soc. 1995; 11:26–8
87. Posthuma WFM, et al. Cardioprotective effect of hormone replacement therapy in postmenopausal women: is the evidence biased? BMJ. 1994;308:1268-9.
88. Barrett-Connor E. Hormone replacement therapy. BMJ. 1998;317:457–61.
89. Panico S, et al. Hormone replacement therapy may not always be the right choice to prevent cardiovascular disease. BMJ. 1996;313:687 (lettera).
90. Panico S, et al. Terapia ormonale sostitutiva e malattie cardiovascolari: popolazioni differenti, rischi differenti. Ann Ist Sup Sanità. 1997:33,2:203-6.
91. Eysenbach G, et al. Empirical studies assessing the quality of health information for consumers on the world wide web: a systematic review. JAMA. 2002; 287:2691–700.
92. Perez-Lopez FR. An evaluation of the contents and quality of menopause information on the World Wide Web. Maturitas. 2005; 49:276–82.
